# Unstable Housing and Mortality Among US Veterans Receiving Dialysis

**DOI:** 10.1001/jamanetworkopen.2023.44448

**Published:** 2023-11-21

**Authors:** Tessa K. Novick, Michael J. Mader, Kirsten L. Johansen, Elizabeth C. Matsui, Elizabeth Montgomery, Elizabeth A. Jacobs, Deidra C. Crews

**Affiliations:** 1 University of Texas at Austin Dell Medical School, Austin; 2 Audie L. Murphy Veterans Affairs Medical Center, San Antonio, Texas; 3 Hennepin Healthcare, Minneapolis, Minnesota; 4 School of Public Health, University of Alabama, Birmingham; 5 Alabama Veterans Affairs Health Care System, Birmingham; 6 Maine Medical Center, Portland; 7 Johns Hopkins University School of Medicine, Baltimore, Maryland

## Abstract

**Importance:**

Housing status is an important health determinant, yet little is known about unstable housing among individuals receiving dialysis.

**Objective:**

To determine factors associated with unstable housing among US veterans receiving dialysis and to estimate the association of unstable housing with risk of death.

**Design, Setting, and Participants:**

This retrospective cohort study used data from the US Veterans Health Administration (VHA) and the US Renal Data System for patients who initiated dialysis between October 1, 2012, and December 31, 2018. Veterans were included if they used VHA outpatient services and completed 1 or more unstable housing screenings within a 3-year period before starting dialysis. Data analysis was conducted from January 24 to June 16, 2023.

**Exposures:**

Unstable housing was defined as self-report of not having stable housing within the past 2 months or having concerns about stable housing in the next 2 months.

**Main Outcomes and Measures:**

The main outcome was all-cause mortality. Characteristics associated with unstable housing at the time of dialysis initiation were examined. The multivariate Fine and Gray cumulative incidence model was used, treating transplant as a competing risk and age as an effect modifier, to examine the risk of death associated with unstable housing.

**Results:**

This study included 25 689 veterans, with a median age of 68 (IQR, 62-74) years. Most participants were men (98%), and more than half (52%) were White. There were 771 veterans (3%) with a positive screen for unstable housing within a 3-year period before starting dialysis. Compared with veterans with stable housing, those with unstable housing were younger (mean [SD] age, 61 [8] vs 68 [10] years), were more likely to be Black (45% vs 32%) or Hispanic (9% vs 7%), and were more likely to start dialysis with a central venous catheter (77% vs 66%), receive in-center hemodialysis (96% vs 91%), and have non-Medicare insurance (53% vs 28%). Factors associated with unstable housing included Hispanic ethnicity, non–arteriovenous fistula vascular access, lack of predialysis nephrology care, and non-Medicare insurance. Veterans with unstable housing had higher all-cause mortality (adjusted hazard ratio [AHR], 1.20 [95% CI, 1.04 to 1.37] for a median age of 68 years), and risks increased with age (*P* = .01 for interaction). In age-stratified analyses, unstable housing was associated with higher mortality among veterans aged 75 to 85 years (AHR, 1.64 [95% CI, 1.18 to 2.28]), but associations were not observed for other age groups.

**Conclusions and Relevance:**

In this cohort study of veterans receiving dialysis, unstable housing experienced before starting dialysis was associated with increased risk of all-cause mortality, and risks increased with age. Further efforts are needed to understand the experiences of older adults with unstable housing and to estimate the scope of unstable housing among all individuals receiving dialysis.

## Key Points

**Question:**

Among US veterans receiving dialysis, is unstable housing associated with mortality and does risk differ according to age?

**Findings:**

In this cohort study of 25 689 veterans receiving dialysis, unstable housing was associated with an increased risk of all-cause mortality, and this risk increased with age.

**Meaning:**

These findings suggest that unstable housing may contribute to socioeconomic disparities in mortality among US veterans receiving dialysis, with older adults being particularly vulnerable.

## Teaser

This cohort study identifies characteristics associated with unstable housing among US veterans receiving dialysis and estimates the association between unstable housing and mortality risk.

## Introduction

Unstable housing is variably defined but includes homelessness and housing that one might lose because it is unaffordable, overcrowded, or dangerous.^1^ There is mounting concern that housing issues may be prominent among individuals receiving dialysis, given increasing housing costs across the US and the job loss and financial resource strain associated with dialysis initiation.^1,2,3^ Unstable housing has the potential to negatively affect nearly every aspect of dialysis care and increase complications, and it limits access to home dialysis modalities and kidney transplantation.^3^ Interventions addressing housing as part of clinical care have the potential to lower risk of mortality.^3,4,5^ However, little is known about unstable housing among individuals receiving dialysis.

Since 2010, the Office of the President of the United States and the US Department of Veterans Affairs have made it a priority to eliminate homelessness among veterans.^6^ Comprehensive efforts have included investing in veteran-specific housing programs that use a housing-first approach, increasing affordable housing for veterans, and funding programs that prevent veterans from becoming homeless.^6^ Screening for unstable housing at US Veterans Health Administration (VHA) clinics began in 2012 as part of these national efforts; a positive screen for unstable housing triggers a timely referral to veteran-specific housing programs.^7^ With this initiative, the number of homeless veterans decreased from 74 087 to 33 129 between 2010 and 2022.^6^

The goals of this study were (1) to identify characteristics associated with unstable housing among veterans receiving dialysis and (2) to evaluate potential associations between unstable housing before dialysis initiation and mortality. There is evidence that socioeconomic disparities in mortality differ according to age for individuals receiving dialysis,^8^ so we sought to examine whether the association between unstable housing and mortality differed according to age. We hypothesized that unstable housing would be associated with increased risk of all-cause mortality and risks would increase with age.

## Methods

### Study Population

This cohort study was approved by the institutional review boards of the University of Texas Health Science Center at San Antonio, Audie L. Murphy Veterans Memorial Hospital, and University of Texas at Austin Dell Medical School. Informed consent was waived in accordance with the Common Rule because data were deidentified. The study followed the Strengthening the Reporting of Observational Studies in Epidemiology (STROBE) reporting guideline.

**Figure 1.**
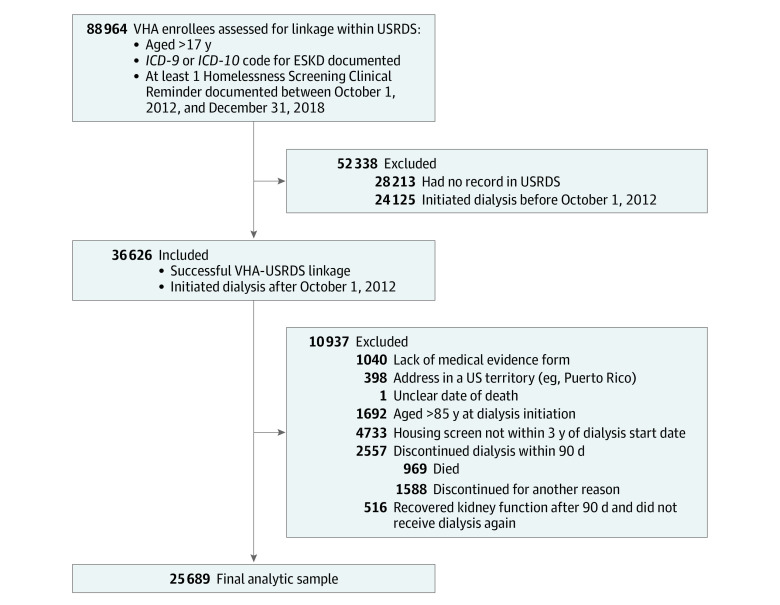
Study Flow Diagram ESKD, end-stage kidney disease; *ICD-9*, *International Classification of Diseases, Ninth Revision*; *ICD-10*, *International Statistical Classification of Diseases and Related Health Problems, Tenth Revision*; USRDS, US Renal Data System; VHA, US Veterans Health Administration.

We developed a retrospective cohort examining veterans who initiated dialysis between October 1, 2012, and December 31, 2018 (Figure 1). Entry into the cohort was defined by the presence or absence of an *International Classification of Diseases, Ninth Revision* (*ICD-9*), or *International Statistical Classification of Diseases and Related Health Problems, Tenth Revision* (*ICD-10*), diagnostic code for end-stage kidney disease and the presence of a screener for unstable housing. The Veterans Information Resource Center identified 36 626 veterans receiving dialysis by linking with US Renal Data System (USRDS) data for veterans who had obtained health care at VHA clinics, were screened for unstable housing, and started dialysis within the designated time period. We chose a start date of October 1, 2012, since that was when housing screening began at VHA locations. We excluded individuals who were not aged between 18 and 85 years at dialysis initiation, had housing screen data that were not within the 3-year period before starting dialysis, were missing the medical evidence form, lived in a US territory (eg, Puerto Rico or Guam), recovered kidney function during the study period, or discontinued dialysis in less than 90 days due to death, kidney transplant receipt, or loss to follow-up (n = 10 937). We excluded individuals aged older than 85 years, given the contribution of age to mortality. In addition, we excluded those who discontinued dialysis in less than 90 days to study associations with unstable housing among individuals with kidney failure instead of acute kidney disease or kidney injury. The final analytic cohort consisted of 25 689 veterans. There were no substantial differences between included and excluded individuals (eTable 1 in Supplement 1).

### Exposure Variable

The exposure of interest was unstable housing within 3 years before dialysis initiation, ascertained from the Corporate Data Warehouse (CDW) Homelessness Screening Clinical Reminder (eAppendix and eTable 2 in Supplement 1).^7,9,10,11,12^ The clinical reminder includes 2 questions: “In the past two months, have you been living in stable housing that you own, rent, or stay in as part of a household?” and “Are you worried or concerned that in the next two months you may not have stable housing that you own, rent or stay in as part of a household?” Development and validation of the clinical reminder was described previously, and the screener has demonstrated good internal consistency (reliability coefficient, 0.85).^12^ The Homelessness Screening Clinical Reminder is administered annually in outpatient primary care and mental health settings.^11^ Our sample included veterans with at least 1 complete housing screen; thus, there was no missingness. A positive screen increases screening frequency to every 6 months, and 3 consecutive negative screens extend screening to every 2 years. Using all available screens within 3 years before dialysis initiation, we defined unstable housing (yes or no) as answering “no” to having stable housing within the past 2 months and/or “yes” to being worried about not having stable housing in the upcoming 2 months.^10^ We used the screen most proximal to the date of dialysis initiation. We did not evaluate housing screens after dialysis initiation.

### Covariates

We combined data from the VHA CDW and USRDS to determine baseline demographic characteristics (eTable 3 in Supplement 1). We obtained information on age, sex (female or male), race and ethnicity (analyzed as mutually exclusive categories of Black, Hispanic, White, or other race or ethnicity [specific categories not available]), and urban or rural residence (urban, rural, or highly rural) from the VHA CDW. Race and ethnicity data were obtained from electronic medical records, which are usually self-report; these data were included due to associations between race and ethnicity and mortality among people receiving dialysis.^13^

We obtained information from the USRDS on year of dialysis initiation, medical insurance at dialysis initiation (Medicaid, Medicare, group health insurance, other coverage, or no coverage), incident vascular access (arteriovenous fistula, central venous catheter, arteriovenous graft, other, or unknown), predialysis nephrology care (yes, no, or unknown), primary cause of kidney failure (hypertension, diabetes, cystic kidney disease, glomerulonephritis, other, or unknown), and incident dialysis modality (in-center hemodialysis, peritoneal dialysis, home hemodialysis, or unknown).

We extracted information on comorbidities from VHA inpatient and outpatient data tables using *ICD-9* and *ICD-10* codes (eTable 4 in Supplement 1). We assigned comorbidities if an individual had 2 outpatient visits or 1 inpatient visit with the relevant diagnostic code at any time before dialysis initiation. We looked back up to 3 years from the dialysis start date for the presence of comorbidities and drug dependence variables.

### Outcome Assessment

Our primary outcome of interest was time to death from 90 days after dialysis initiation. We obtained mortality, censoring and competing events, and associated dates from the VHA CDW and USRDS files. We followed participants from 90 days after dialysis initiation until death, transplantation, discontinuation of dialysis, loss to follow-up, or end of the follow-up period (December 31, 2018). Information on transplantation, recovery of kidney function, discontinuation of dialysis, and loss to follow-up was obtained from the USRDS.

### Statistical Analysis

We compared baseline characteristics according to unstable housing status, and we present data as means (SDs) or medians (IQRs) for continuous variables and numbers or percentages for categorical variables. We used logistic regression, with unstable housing as the outcome and all covariates included in one model, to assess correlates of unstable housing within 3 years before dialysis initiation.

We estimated the overall cumulative incidence of mortality for veterans with and without unstable housing. We used the Fine and Gray^12^ cumulative incidence method to estimate adjusted associations between unstable housing within 3 years before dialysis start and mortality. The Fine and Gray method is conceptually similar to the Cox proportional hazards regression method but allows for competing risks. We incrementally adjusted for the following covariates based on the literature and theoretical considerations: model 1 adjusted for age; model 2 additionally adjusted for sex, race and ethnicity, year of dialysis initiation, and predialysis nephrology care; and model 3 additionally adjusted for dialysis modality, cause of kidney failure, vascular access, medical insurance, urban or rural residence, and comorbidities. We included an age-squared term in all models to account for the nonlinear effects of age.

We tested the proportional hazards assumption using Schoenfeld residuals, which was met for all covariates in models 1 and 2, including age.^14,15^ The proportional hazards assumption was not met for some of the comorbidities in model 3, so the variables that did not meet the assumption were instead adjusted for as stratified variables.^14,15^

We tested for effect modification by age by including an interaction term between unstable housing × age in models 2 and 3. We tested for effect modification by race and ethnicity by including interaction terms for unstable housing × race and ethnicity and for unstable housing × age × race and ethnicity in the overall survival analysis.

We stratified the cohort by age (<50, 50-64, 65-74, or 75-85 years) and compared survival between those with and without unstable housing using the Fine and Gray method. We adjusted for the factors previously listed (minus the unstable housing × age interaction term).

In all models, we treated the small numbers of discontinuation and loss to follow-up as censoring events equivalent to the end of the study period, using the date of last known dialysis as the censor date. However, we treated transplantation as a competing risk. Individuals who experience unstable housing are often not considered eligible for kidney transplantation. Thus, the Cox model estimates the risk of death if individuals with and without unstable housing underwent transplantation at equal rates, whereas the Fine and Gray competing risk model better estimates the risk of death on dialysis given the disparity in access to transplantation.^16^ To confirm whether we should treat transplantation as a competing risk, we estimated associations between unstable housing and transplantation, with death as a competing risk, and adjusted for age, sex, race and ethnicity, year of dialysis initiation, and predialysis nephrology care.

We conducted several sensitivity analyses as follows. We repeated the overall population analysis without the interaction term between unstable housing × age. We repeated the primary analysis and analyzed age as a categorical variable (<50, 50-64, 65-74, or 75-85 years) instead of a continuous variable. We repeated the primary analysis and included individuals aged older than 85 years. We limited the sample to participants who did not have discrepancies between USRDS and VHA data on date of death and repeated the primary analysis (n = 25 617). Finally, we limited the sample to participants who were screened for unstable housing within 1 year of dialysis initiation and repeated the primary analysis (n = 23 644).

Due to the availability of information from 2 data sets and because we excluded patients with missing medical evidence forms, we had only 15 individuals with missing data (in the fields of incident dialysis modality and rurality).

Statistical significance was set at *P* < .05, with 2-tailed tests. Data analysis was conducted from January 24 to June 16, 2023, using SAS 9.4/STAT, version 15.3 (SAS Institute Inc).

## Results

### Baseline Characteristics of Individuals With Unstable Housing

This study included 25 689 veterans with a median age of 68 (IQR, 62-74) years. Men comprised 98% of the study population and women comprised 2%. Of the included patients, 32% were Black, 7% were Hispanic, 52% were White, and 10% were of other race or ethnicity. There were 771 veterans (3%) with a positive screen for unstable housing within a 3-year period before starting dialysis. Compared with veterans without indication of unstable housing, those with unstable housing were younger (mean [SD] age, 61 [8] vs 68 [10] years); they were also more likely to be women (5% vs 2%), to be Black (45% vs 32%) or Hispanic (9% vs 7%), and to live in urban areas (82% vs 69%) (Table 1). They were less likely to have predialysis nephrology care (16% vs 23%), and they were more likely to start dialysis with a central venous catheter (77% vs 66%), receive in-center hemodialysis (96% vs 91%), and have non-Medicare insurance (53% vs 28%). Covariates associated with higher odds of unstable housing included younger age, female vs male sex, Hispanic vs non-Hispanic ethnicity, lack of predialysis nephrology care, primary insurance (non-Medicare vs Medicare), chronic obstructive pulmonary disease, alcohol dependence, and drug dependence. Having an arteriovenous fistula vs a central venous catheter and residing in a rural vs urban area were associated with lower odds of unstable housing.

**Table 1.** Baseline Study Population Characteristics According to Unstable Housing Status and Factors Associated With Unstable Housing^a^

Characteristic	All patients (N = 25 689)	Patients with unstable housing (n = 771)	Patients with stable housing (n = 24 918)	Logistic regression of factors assocated with unstable housing, OR (95% CI) (n = 25 674)^b^
Age group, y				
<50	1048 (4)	54 (7)	994 (4)	1.22 (1.13 to 1.32)
50-64	7214 (28)	447 (58)	6767 (27)	
65-74	11 405 (44)	224 (29)	11 181 (45)	
75-85	6022 (23)	46 (6)	5976 (24)	
Sex				
Female	598 (2)	37 (5)	561 (2)	1.52 (1.06 to 2.17)
Male	25 091 (98)	734 (95)	24 357 (98)	1 [Reference]
Race and ethnicity				
Black	8108 (32)	344 (45)	7764 (31)	1.13 (0.94 to 1.36)
Hispanic	1751 (7)	69 (9)	1682 (7)	1.34 (1.01 to 1.78)
White	13 385 (52)	263 (34)	13 122 (53)	1 [Reference]
Other^c^	2445 (10)	95 (12)	2350 (9)	1.32 (1.03 to 1.70)
Year of dialysis initiation				
2012	555 (2)	8 (1)	547 (2)	0.35 (0.17 to 0.71)
2013	3629 (14)	79 (10)	3550 (14)	0.62 (0.46 to 0.82)
2014	4204 (16)	148 (19)	4056 (16)	1 [Reference]
2015	4690 (18)	189 (25)	4501 (18)	1.24 (1.07 to 1.56)
2016	4592 (18)	131 (17)	4461 (18)	0.87 (0.68 to 1.12)
2017	4539 (18)	129 (17)	4410 (18)	0.87 (0.68 to 1.12)
2018	3480 (14)	87 (11)	3393 (14)	0.79 (0.60 to 1.04)
Predialysis nephrology care				
Yes	18 052 (70)	459 (60)	17 593 (71)	1 [Reference]
No	4210 (16)	179 (23)	4031 (16)	1.29 (1.07 to 1.56)
Unknown	3427 (13)	133 (17)	3294 (13)	1.30 (1.06 to 1.60)
Cause of kidney failure				
Diabetes	13 657 (53)	400 (52)	13 257 (53)	1 [Reference]
Cystic kidney disease	455 (2)	15 (0)	440 (2)	1.14 (0.65 to 2.01)
Hypertension	7305 (28)	211 (27)	7094 (28)	0.99 (0.81 to 1.21)
Glomerulonephritis	1507 (6)	60 (8)	1447 (6)	1.20 (0.88 to 1.65)
Other	2385 (9)	70 (9)	2315 (9)	1.04 (0.78 to 1.38)
Unknown	380 (1)	15 (2)	365 (1)	1.23 (0.70 to 2.14)
Vascular access at initiation				
Central venous catheter	16 993 (66)	593 (77)	16 400 (66)	1 [Reference]
Arteriovenous fistula	5971 (23)	122 (16)	5849 (23)	0.70 (0.57 to 0.86)
Arteriovenous graft	682 (3)	23 (3)	659 (3)	1.01 (0.65 to 1.56)
Other	51 (0)	2 (0)	49 (0)	0.90 (0.21 to 3.95)
Unknown	1992 (8)	31 (4)	1961 (8)	0.54 (0.08 to 3.65)
Incident dialysis modality				
In-center hemodialysis	23 482 (91)	739 (96)	22 743 (91)	1 [Reference]
Home hemodialysis	199 (1)	0	199 (1)	NA
Peritoneal dialysis	2007 (8)	32 (4)	1975 (8)	1.03 (0.16 to 6.76)
Medical insurance				
Medicare	17 913 (70)	337 (44)	17 576 (71)	1 [Reference]
Medicaid	1138 (4)	84 (11)	1054 (4)	2.15 (1.65 to 2.79)
Private	872 (3)	20 (3)	852 (3)	0.73 (0.46 to 1.18)
Other	5401 (21)	302 (39)	5099 (21)	1.61 (1.35 to 1.91)
No coverage	365 (1)	28 (4)	337 (1)	1.93 (1.27 to 2.93)
Residence				
Urban	17 771 (69)	629 (82)	17 128 (69)	1 [Reference]
Rural	7687 (30)	134 (17)	7553 (30)	0.60 (0.49 to 0.73)
High rural	231 (1)	8 (1)	223 (1)	1.47 (0.71 to 3.05)
Comorbidity				
Atherosclerotic heart disease	13 269 (52)	387 (50)	12 882 (52)	1.04 (0.88 to 1.22)
Congestive heart failure	12 265 (48)	435 (56)	11 830 (47)	1.16 (0.98 to 1.36)
Chronic obstructive pulmonary disease	6301 (25)	231 (30)	6070 (24)	1.20 (1.00 to 1.43)
Cerebrovascular disease	4629 (18)	159 (21)	4470 (18)	1.07 (0.89 to 1.29)
Cancer	5302 (21)	137 (18)	5165 (21)	0.95 (0.78 to 1.16)
Diabetes	19 235 (75)	580 (75)	18 655 (75)	1.09 (0.88 to 1.35)
Hypertension	24 881 (97)	755 (98)	24 126 (97)	0.85 (0.51 to 1.44)
Peripheral vascular disease	6728 (26)	217 (28)	65,11 (26)	1.02 (0.85 to 1.21)
Posttraumatic stress disorder	3859 (15)	163 (21)	3696 (15)	1.14 (0.94 to 1.37)
Alcohol dependence	2982 (12)	232 (30)	2750 (11)	1.53 (1.27 to 1.85)
Drug dependence	2781 (11)	262 (34)	2519 (10)	2.03 (1.69 to 2.45)
Nicotine dependence	9959 (39)	419 (54)	9540 (38)	1.08 (0.91 to 1.27)

Abbreviations: NA, not applicable; OR, odds ratio.

^a^ Unless indicated otherwise, values are presented as No. (%).

^b^ This analysis had 15 fewer participant records because 1 patient had an unknown value for incident dialysis modality and 14 had unknown values for rurality.

^c^ Specific categories were not available.

### Population-Based Survival Analysis

**Figure 2.**
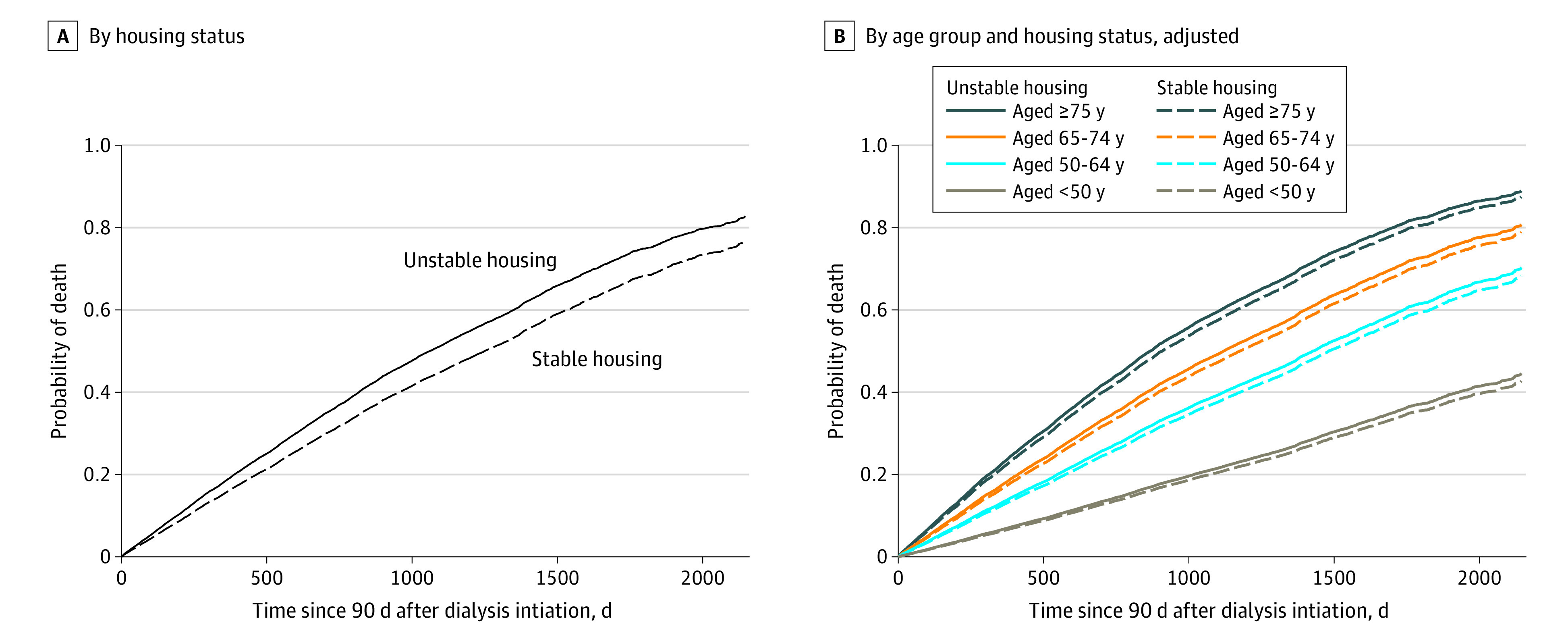
Deaths Among Veterans With and Without Unstable Housing A, Cumulative incidence of deaths among veterans by housing status. B, Cumulative incidence of deaths among veterans by age group and housing status. Data in panels A and B were adjusted for sex, race and ethnicity, year of dialysis initiation, predialysis nephrology care, incident dialysis modality, cause of kidney failure, incident vascular access, primary insurance, urban or rural residence, and comorbidities. Data in panel A were additionally adjusted for age, age-squared, and age × unstable housing. Curves are based on a median age of 68 years, year 2014, and the most common value for all other covariates.

Among the 25 689 veterans with incident kidney failure receiving dialysis, 9435 (37%) died, 767 (3%) received kidney transplantation, and 15 487 (60%) were administratively censored (219 due to discontinuation or loss to follow-up, and the remainder at the end of follow-up; eTable 5 in Supplement 1). The unadjusted cumulative incidence of death was 68%. After multivariable adjustment, veterans with an indicator of unstable housing had higher risk of death compared with veterans with stable housing (adjusted hazard ratio [AHR], 1.20 [95% CI, 1.04 to 1.37] for a median age of 68 years; *P* = .03) (Figure 2 and Table 2). The risk of death associated with unstable housing increased with age (eTable 6 in Supplement 1 presents AHRs for unstable housing at various ages due to interaction). There was no interaction between unstable housing and race and ethnicity (Black: AHR, −0.32 [95% CI, −2.50 to 1.85]; Hispanic: AHR, 0.81 [95% CI, −5.70 to 4.07]; or other: AHR, 0.47 [95% CI, −2.34 to 3.23]; *P* = .97 for interaction) or between unstable housing, race and ethnicity, and age (Black: AHR, 0.01 [95% CI, −0.03 to 0.04]; Hispanic: AHR, 0.01 [95% CI, −0.07 to 0.08]; or other: AHR, −0 [−0.05 to 0.04]; *P* = .98 for interaction).

**Table 2.** Hazard of All-Cause Mortality Among Veterans With Unstable Housing vs Stable Housing, Overall and Stratified by Age Group^a^

Age group, y	Subdistribution HR of all-cause mortality (95% CI)
**Overall (N = 25 689)^b^**	
Model 1	1.14 (1.01 to 1.29)
Model 2	
62 (25th Percentile)	1.12 (0.98 to 1.28)
68 (Median)	1.24 (1.08 to 1.43)
74 (75th Percentile)	1.38 (1.14 to 1.68)
Model 3	
62 (25th Percentile)	1.06 (0.93 to 1.21)
68 (Median)	1.20 (1.04 to 1.37)
74 (75th percentile)	1.35 (1.11 to 1.64)
**<50 (n = 1048)^c^**	
Model 1	1.09 (0.50 to 2.39)
Model 2	1.14 (0.51 to 2.58)
Model 3	1.30 (0.59 to 2.88)
**50-64 (n = 7214)^d^**	
Model 1	1.06 (0.89 to 1.26)
Model 2	1.06 (0.88 to 1.27)
Model 3	1.00 (0.84 to 1.21)
**65-74 (n = 11 405)^e^**	
Model 1	1.17 (0.94 to 1.46)
Model 2	1.22 (0.97 to 1.52)
Model 3	1.18 (0.94 to 1.47)
**75-85 (n = 6022)^f^**	
Model 1	1.55 (1.13 to 2.12)
Model 2	1.60 (1.16 to 2.20)
Model 3	1.64 (1.18 to 2.28)

Abbreviation: HR, hazard ratio.

^a^ Model 1 was adjusted for unstable housing, age, and age-squared. Model 2 was adjusted as for model 1 and race, ethnicity, year of dialysis initiation, predialysis nephrology care, and age × unstable housing. Model 3 was adjusted as for model 2 and incident dialysis modality, cause of kidney failure, incident vascular access, primary insurance, urban/rural, and comorbidities.

^b^ Includes 9435 deaths, 767 transplants, and 15 487 censored. The age × unstable housing interaction term was included in the overall analysis but was not included in the age-stratified models. For all analyses, we followed participants from the day of dialysis initiation until death or censoring events, including loss to follow-up or end of the follow-up period (December 31, 2018).

^c^ Includes 139 deaths, 159 transplants, and 750 censored.

^d^ Includes 2147 deaths, 305 transplants, and 4762 censored.

^e^ Includes 4283 deaths, 274 transplants, and 6848 censored.

^f^ Includes 2866 deaths, 29 transplants, and 3127 censored.

### Stratified Analysis

Among the 1048 veterans aged younger than 50 years with incident kidney failure receiving dialysis, 139 (13%) died, 159 (15%) received kidney transplantation, and 750 (72%) were censored. After multivariable adjustment, veterans with unstable housing had numerically increased risk of death compared with their counterparts with stable housing but the findings were not statistically significant (AHR, 1.30 [95% CI, 0.59 to 2.88]) (Table 2).

Among the 7214 patients aged 50 to 64 years with incident kidney failure receiving dialysis, 2147 (30%) died, 305 (4%) received kidney transplantation, and 4762 (66%) were censored. After multivariable adjustment, there was no association between unstable housing and death (AHR, 1.00 [95% CI, 0.84 to 1.21]).

Among the 11 405 patients aged 65 to 74 years with incident kidney failure receiving dialysis, 4283 (38%) died, 274 (2%) received kidney transplantation, and 6848 (60%) were censored. After multivariable adjustment, veterans with unstable housing had numerically increased risk of death compared with their counterparts with stable housing, but these findings were not statistically significant (AHR, 1.18 [95% CI, 0.94 to 1.47]).

Among the 6022 patients aged 75 to 85 years with incident kidney failure receiving dialysis, 2866 (48%) died, 29 (0.5%) received kidney transplantation, and 3127 (52%) were censored. After multivariable adjustment, individuals with unstable housing had a 64% higher risk of death compared with their counterparts with stable housing (AHR, 1.64 [95% CI, 1.18 to 2.28]).

### Sensitivity Analysis

Veterans with unstable housing had a 70% lower risk of transplantation, supporting the need to address transplantation as a competing risk (AHR, 0.30 [95% CI, 0.17 to 0.55]). The overall risk of death associated with unstable housing was similar when the analysis was repeated without the interaction for unstable housing × age (eTable 7 in Supplement 1). Findings were similar in sensitivity analyses in which (1) we included individuals aged older than 85 years, (2) we analyzed age as a categorical variable instead of a continuous variable, (3) we limited the sample to participants who did not have discrepancies between USRDS and VHA data on date of death, and (4) we restricted the sample to veterans who were screened within 1 year before starting dialysis (eTables 8, 9, 10, and 11 in Supplement 1, respectively).

## Discussion

Among a nationwide sample of 25 689 veterans receiving dialysis who were screened for unstable housing at outpatient VHA locations, we observed that unstable housing within 3 years before starting dialysis was associated with higher all-cause mortality. Risk of death associated with unstable housing increased with age, with veterans aged 75 to 85 years having the highest risk.

Our findings agree with existing literature on mortality risks associated with housing issues in the general population. For example, Roncarati et al^17^ compared all-cause mortality between adults experiencing homelessness in Boston and the general Massachusetts population and found that mortality among homeless adults living in shelters and unsheltered locations was 3-fold and 10-fold higher, respectively. Our findings are also consistent with other studies reporting that housing issues late in life are associated with increased mortality risk. For example, in a cohort study of 450 adults aged older than 50 years experiencing homelessness, having a first episode of homelessness in late life was associated with higher mortality.^18^

In the general population, unstable housing is thought to affect outcomes by acting as a barrier to seeking and obtaining health care. When individuals experience unstable housing, their housing may be prioritized over their health. High housing costs limit the availability of money for food, medications, and transportation. Limited financial resources may contribute to difficulty in scheduling and attending nephrology appointments or obtaining arteriovenous fistulas placed for individuals with advanced chronic kidney disease. The demands associated with dialysis may limit patients’ ability to work, further limiting financial resources, and increasing stress. Stress is postulated to enhance activation of the sympathetic nervous system, the renin-angiotensin-aldosterone system, and the hypothalamic-pituitary-adrenal axis and to contribute to risk of cardiovascular events.^19,20,21,22,23,24,25,26,27^

Documented income disparities in survival among individuals receiving dialysis are thought to be partially driven by lack of insurance.^28,29,30^ Our findings contribute to the literature by highlighting the potential role of unstable housing as a driver of socioeconomic disparities independent of access to health care, since veterans have universal health care. We also observed that unstable housing was associated with a lack of predialysis nephrology care, non–fistula incident vascular access, and in-center hemodialysis as the initial dialysis modality, suggesting that housing issues fragment the transition from chronic kidney disease to dialysis initiation, despite access to care.

### Strengths and Limitations

The strengths of this study include its large sample size inclusive of a nationwide sample and identification of a risk factor for mortality among individuals receiving dialysis. Our analysis of subgroups allowed us to identify older adults as a population that is particularly vulnerable to the adverse effects of unstable housing.

This study also has several limitations. Veterans had to receive care at an outpatient VHA clinic to be screened for unstable housing, and the Homelessness Screening Clinical Reminder was not uniformly rolled out at all VHA sites at the same time, potentially limiting the representativeness of our sample. Individuals who seek care at VHA clinics and are screened for unstable housing may differ from veterans who seek care elsewhere, introducing possible selection bias. However, we observed no statistically significant differences between veterans who were included or excluded. The association between a positive unstable housing screen and mortality may be different for veterans than nonveterans due to the availability of unique housing programs within the VHA, limiting generalizability to the civilian population. We did not have time-updated information on unstable housing or comorbidities.

Depending on when the Homelessness Screening Clinical Reminder was administered and because housing status often changes, we may have captured individuals’ past (within the prior 3 years) or general experience rather than their current experience. Underrepresentation of younger individuals in the data and use of a linear unstable housing × age interaction term may have affected estimations for younger age groups. We did not have nuanced information about participant race and ethnicity.

## Conclusions

In this cohort study of US veterans receiving dialysis, unstable housing experienced before dialysis initiation was associated with increased risk of all-cause mortality. Risks increased with age and were highest among older veterans. Further efforts are needed to understand the experiences of older adults with unstable housing and to estimate the scope of unstable housing among all individuals receiving dialysis.

## Notes

Supplement 1. **eTable 1.** Baseline Study Population Characteristics: Included vs Excluded Participants **eAppendix.** Exposure Definition **eTable 2.** Exposure Definition **eTable 3.** Data Sources **eTable 4.** *International Classification of Diseases* (*ICD*) Codes for Covariables **eTable 5**. Outcomes at Various Time Points for Individuals With and Without Unstable Housing **eTable 6.** Hazard of All-Cause Mortality Associated With Unstable Housing for Various Ages **eTable 7.** Sensitivity Analysis: Analysis Without Age × Unstable Housing Interaction Term **eTable 8.** Sensitivity Analysis: Including Individuals Aged Older Than 85 Years **eTable 9.** Sensitivity Analysis: Analysis With Age as a Categorical Variable **eTable 10.** Sensitivity Analysis: Sample Limited to Participants Who Did Not Have Discrepancies Between US Renal Data System and Veterans Health Administration Data on Date of Death **eTable 11.** Sensitivity Analysis: Sample Limited to Participants Who Were Screened for Unstable Housing Within 1 Year Before Dialysis Initiation

Supplement 2.
Data Sharing Statement
