# Supplementary material for: Unstable Housing and Mortality Among US Veterans Receiving Dialysis
Source: JAMA Netw Open. 2023 Nov 21;6(11):e2344448. doi: 10.1001/jamanetworkopen.2023.44448 (PMC10663965; doi:10.1001/jamanetworkopen.2023.44448)
Supplement: Supplement 1. — eTable 1. Baseline Study Population Characteristics: Included vs Excluded Participants eAppendix. Exposure Definition eTable 2. Exposure Definition eTable 3. Data Sources eTable 4. International Classification of Diseases (ICD) Codes for Covariables eTable 5. Outcomes at Various Time Points for Individuals With and Without Unstable Housing eTable 6. Hazard of All-Cause Mortality Associated With Unstable Housing for Various Ages eTable 7. Sensitivity Analysis: Analysis Without Age × Unstable Housing Interaction Term eTable 8. Sensitivity Analysis: Including Individuals Aged Older Than 85 Years eTable 9. Sensitivity Analysis: Analysis With Age as a Categorical Variable eTable 10. Sensitivity Analysis: Sample Limited to Participants Who Did Not Have Discrepancies Between US Renal Data System and Veterans Health Administration Data on Date of Death eTable 11. Sensitivity Analysis: Sample Limited to Participants Who Were Screened for Unstable Housing Within 1 Year Before Dialysis Initiation [file jamanetwopen-e2344448-s001.pdf]

Supplementary Online Content

Novick TK, Mader MJ, Johansen KL, et al. Unstable housing and mortality among US veterans receiving dialysis. *JAMA Netw Open*. 2023;6(11):e2344448. doi:10.1001/jamanetworkopen.2023.44448

eTable 1. Baseline Study Population Characteristics: Included vs Excluded Participants
eAppendix. Exposure Definition
eTable 2. Exposure Definition
eTable 3. Data Sources
eTable 4. *International Classification of Diseases* (*ICD*) Codes for Covariables
eTable 5. Outcomes at Various Time Points for Individuals With and Without Unstable Housing
eTable 6. Hazard of All-Cause Mortality Associated With Unstable Housing for Various Ages
eTable 7. Sensitivity Analysis: Analysis Without Age × Unstable Housing Interaction Term
eTable 8. Sensitivity Analysis: Including Individuals Aged Older Than 85 Years
eTable 9. Sensitivity Analysis: Analysis With Age as a Categorical Variable
eTable 10. Sensitivity Analysis: Sample Limited to Participants Who Did Not Have Discrepancies Between US Renal Data System and Veterans Health Administration Data on Date of Death
eTable 11. Sensitivity Analysis: Sample Limited to Participants Who Were Screened for Unstable Housing Within 1 Year Before Dialysis Initiation

This supplementary material has been provided by the authors to give readers additional information about their work.

© 2023 Novick TK et al. *JAMA Network Open*.

eTable 1. Baseline Study Population Characteristics: Included vs Excluded Participants

Characteristic	Included	Excluded
N	25,689	10,937
Age group, years, n (%)		
<50	1,048 (4.08)	436 (3.99)
50-64	7,214 (28.08)	2,593 (23.71)
65-74	11,405 (44.40)	3,744 (34.23)
≥75	6,022 (23.44)	4,164 (38.07)
Sex, n (%)		
Female	598 (2.30)	213 (1.95)
Male	25,091 (97.67)	10,724 (98.05)
Race and Ethnicity, n (%)		
Black	8,108 (31.56)	3,053 (27.91)
Hispanic	1,751 (6.82)	961 (8.79)
Other	2,445 (9.52)	960 (8.78)
White	13,385 (52.10)	5,963 (54.52)
Year of dialysis initiation, n (%)		
2012	555 (2.16)	827 (7.56)
2013	3,629 (14.13)	2,019 (18.46)
2014	4,204 (16.36)	1,544 (14.12)
2015	4,690 (18.26)	1,472 (13.46)
2016	4,592 (17.88)	1,505 (13.76)
2017	4,539 (17.67)	1.352 (12.36)
2018	3,480 (13.55)	2,218 (20.28)
Cause of Kidney Failure, n (%)		
Cystic kidney disease	455 (1.77)	169 (1.55)
Diabetes	13,657 (53.16)	4,498 (41.13)
Hypertension	7,305 (28.44)	3,267 (29.87)
GN	1,507 (5.87)	627 (5.73)
Other	2,385 (9.28)	1,323 (12.10)
Unknown	380 (1.48)	1,053 (9.63)
Incident dialysis modality, n (%)		
In-center Hemodialysis	23,482 (91.41)	9,905 (90.56)
Home Hemodialysis	199 (0.77)	73 (0.67)
Peritoneal dialysis	2,007 (7.81)	808 (7.39)
Unknown	1 (0.0)	151 (1.38)

© 2023 Novick TK et al. *JAMA Network Open*.

eAppendix. Exposure Definition

We defined unstable housing using the data collected from the Homelessness Screening Clinical Reminder. The Homelessness Screening Clinical Reminder is embedded within the Veterans Health Administration (VHA) electronic medical record, Computerized Patient Record System. It is placed on the cover sheet for all patients at every VA Medical Center. Any staff member can perform the screen. The screen is administered annually at outpatient clinics. Veterans that screen positive (currently homeless or being in imminent danger of homelessness) are then screened every 6 months. Indicating that someone is a resident of a long-term care facility sets the frequency to every 2 years. Once a Veteran has screened negative for homelessness three (3) consecutive times, the frequency changes to every 2 years. The reminder excludes Veterans if a visit is found within the last 6 months in a clinic associated with any of the stop codes indicative of homeless services.

The Homelessness Screening Clinical Reminder started in late 2012 after the President of the United States and Secretary of the Department of Veterans Affairs made it a priority to eliminate homelessness in the Veteran population. There are 130 health care systems (HCS) within the VHA, and not all HCS began using the screen at the same time. Each HCS had the option to add questions to their screen, if appropriate. In order for a Veteran to have a screen they would have attended an appointment at a VHA facility that was administering the Homelessness Screening Clinical Reminder.

VHA data is stored in the Corporate Data Warehouse as health factors. There are no enterprise-wide naming conventions, formatting, or standards requirements for

© 2023 Novick TK et al. *JAMA Network Open*.

health factors, and no functionality exists to designate, for example, a health factor as "national". Someone at the national level might suggest for individual HCS's to start using and document health factors, but each HCS has some flexibility to decide if and when to start using them, and what specific language will be recorded for the health factor.

We used database queries to investigate which health factor statements for the Homelessness Screening Clinical Reminder have been used over time and at which locations. We found 43 different statements associated with the health factor category called "Homelessness Screening," although many of the statements were associated with food insecurity. The time span of interest was from Oct 2012 to Sept 2019. Below is a summary of the Homelessness Screening health factors identified specifically related to unstable housing, and whether we included them in our exposure definition.

© 2023 Novick TK et al. *JAMA Network Open*.

eTable 2. Exposure Definition

HCS	Time in Use	Clinical Reminder	Health Factor	Whether/how Included in Definition of Unstable housing
All 130 HCSs	October 2012 to present	1. For the past 2 months, have you been living in stable housing that you own, rent, or stay in as part of a household?<br>2. Are you worried or concerned that in the next 2 months you may not have stable housing that you own, rent, or stay in as part of a household?<br>3. Screen not performed: Already receiving homeless services or assistance<br>4. Screen not performed: Long term resident of nursing home/LTC Facility<br>5. Screen not performed: Declines screening at this time<br>6. Screen not performed: Veteran/Caregiver unable to answer<br>7. Would you like to talk more about your housing situation?	1a. Negative - has stable housing<br>1b. Positive - has no stable housing<br>2a. Positive - has worries about housing<br>2b. Negative - has no housing concerns<br>3. Already receiving assistance with housing<br>4. Nursing home resident<br>5. Declines homeless screen<br>6. Unable to perform homeless screen<br>7a. Referred to homeless program<br>7b. Declined homeless referral<br>7c. Referred to social work<br>7d. Declines social work referral	Included:<br>- housing secure: 1a, 2b, 4<br>- housing insecure: 1b, 2a<br><br>Not included:<br>-3, 5-7
HCS 585 only	April 2015 to present	Same questions as in first row, with additional possible responses	2c. Housing concern no<br>2d. Housing concern yes declines referral	Included<br>- housing secure: 2c<br>- housing insecure: 2d, 2e

© 2023 Novick TK et al. *JAMA Network Open*.

			2e. Housing concern yes referred	
HCS 646 only	Oct 2012 - Aug 2016	Same questions as in first row, with additional possible responses	1c. Homeless currently<br>2c. Homeless future housing concern<br>7e. Homeless referral declined	Included<br>- housing secure:<br>- housing insecure: 1c, 2c
HCS 691 only	Sept 2013 - present	Same questions as in first row, with additional response	1c. Homeless on admission	Included as housing insecure

© 2023 Novick TK et al. *JAMA Network Open*.

eTable 3. Data Sources

Variable	Data Source
Age/Date of birth	Primary source: VHA CDW<br>Secondary source: USRDS<br><br>Note: When there were discrepancies between the two data sources we used the CDW. Approximately 1% of values did not match between the two sources; approximately 60% of those had age discrepancies between 3 months and 3 years and the rest were >3 years. We used the secondary source when the date of birth listed in the primary source was clearly incorrect (e.g., if it was after the date of death).
Sex/gender	Primary source: VHA CDW<br>Secondary source: USRDS<br><br>Note: approximately 0.4% of participants had mismatched sex/gender. We used the primary source, but used the secondary source if sex was missing in the CDW.
Race and Ethnicity	Primary source: VHA CDW<br>Secondary source: USRDS<br><br>Note: Approximately 18% of participants had mis-matched data on race/ethnicity (primarily due to being listed as “unknown” in one source). There were 950 participants with discrepancies between Black/white race, and 750 with discrepancies between Hispanic/non-Hispanic status. Both data sources are theoretically self-report. We used VHA data unless race or ethnicity were unknown, in which case we used USRDS.
Year of dialysis initiation	USRDS<br><br>Note: For participants with more than one start date, we used the first start date for which they were on dialysis for >90 days (e.g. when someone was on dialysis for 60 days, discontinued, then started again 2 years later for several years, we used the second date).
Pre-dialysis nephrology care	USRDS

© 2023 Novick TK et al. *JAMA Network Open*.

Cause of Kidney Failure	USRDS
Vascular Access at Initiation	USRDS
Incident dialysis modality	USRDS<br><br>Note: We used the first modality for the first dialysis period that lasted at least 90 days.
Medical Insurance	USRDS
Residence (Urban/rural/high rural)	VHA CDW
Comorbidities	VHA CDW
Date of death	Primary: VHA CDW<br>Secondary: USRDS<br><br>Note: We used USRDS when the date of death was missing from the CDW. When the two sources listed different dates, we looked for evidence of care in the CDW to try to determine the correct date of death (e.g. if there was care documented after the USRDS date of death we used the CDW date of death).
Date of kidney transplantation	USRDS

Abbreviations: VHA – Veterans Health Administration; CDW – Corporate Data Warehouse; USRDS – United States Renal Data System

© 2023 Novick TK et al. *JAMA Network Open*.

eTable 4. *International Classification of Diseases* (*ICD*) Codes for Covariables

Covariable	ICD 10 code	ICD 9 code
Atherosclerotic Heart Disease	I21.x, I24.x, I25.x	410.x, 411.x, 412, 414.x
Cardiac failure	I09.81, I11.0, I13.x, I25.5, I42.x, I43, 150.x	398.91, 402.x, 404.x, 425.x, 428.x
Peripheral vascular disease	170.x	440.x, 443.x
Cerebral vascular disease	G45.x, G46.x, I63.x, I67.x, I68.x	362.34, 433.x, 434.x, 435.x, 436, 437.x,
Hypertension	I10.x to I15.x	401.x to 405.x
Diabetes	E10.x to E11.x	250.x
Cancer	C00.x to C43.x, C45.x to C76.x, C7A.x, C80.1, C81.x to C96.	140.x to 172.x, 174.x to 195.x, 199.x, 200.x to 209.2x
Chronic obstructive pulmonary disease	J40.x to J44.x, J47.x	490.x to 492.x, 494.x to 496.x
History of drug dependence	F11.x to F19.x	304.0x to 304.9x, 305.8x, 305.9x
History of alcohol dependence	F10.x	303.9x, 305.0x
Post-traumatic stress disorder	F43.1x	309.81
Tobacco use	Z72.0, Z87.891, F17.x	V15.82, 305.1

© 2023 Novick TK et al. *JAMA Network Open*.

eTable 5. Outcomes at Various Time Points for Individuals With and Without Unstable Housing

Time from 90 days after dialysis initiation	0 days	500 days	1000 days	1500 days	2000 days
**Unstable Housing**					
At Risk	771	500	273	93	11
Deceased since previous time	0	120	89	33	10
Transplanted since previous time	0	2	4	4	0
Censored since previous time	0	149	134	143	72
**Stable Housing**					
At Risk	24,918	14,853	7,605	2,887	444
Deceased since previous time	0	4,312	2,849	1,468	532
Transplanted since previous time	0	301	256	143	53
Censored since previous time	0	5,452	4,143	3,107	1,858

© 2023 Novick TK et al. *JAMA Network Open*.

eTable 6. Hazard of All-Cause Mortality Associated With Unstable Housing for Various Ages

Age Group	Sub-distribution Hazard Ratios of All-cause Mortality for Unstable Housing vs Stable Housing (95% Confidence Interval)	
	Model 1	Model 2
Age = 40	0.75 (0.51 - 1.12)	0.69 (0.46 - 1.02)
Age = 45	0.82 (0.60 - 1.14)	0.76 (0.55 - 1.05)
Age = 50	0.90 (0.70 - 1.16)	0.84 (0.65 - 1.08)
Age = 55	0.99 (0.81 - 1.20)	0.92 (0.76 - 1.12)
Age = 60	1.08 (0.93 - 1.25)	1.02 (0.88 - 1.18)
Age = 65	1.18 (1.04 - 1.34)	1.13 (0.99 - 1.28)
Age = 70	1.29 (1.11 - 1.50)	1.25 (1.07 - 1.45)
Age = 75	1.41 (1.15 - 1.73)	1.38 (1.12 - 1.69)
Age = 80	1.54 (1.18 - 2.01)	1.52 (1.16 - 1.99)

Model 1: adjusted for age, age-squared, sex, race, ethnicity, year of dialysis initiation, pre-dialysis nephrology care, and age × unstable housing interaction term

Model 2: model 1 + incident dialysis modality, cause of kidney failure, incident vascular access, primary insurance, urban/rural, and comorbidities

© 2023 Novick TK et al. *JAMA Network Open*.

eTable 7. Sensitivity Analysis: Analysis Without Age × Unstable Housing Interaction Term

Model	Sub-distribution Hazard Ratio of All-cause Mortality for Unstable Housing vs Stable Housing (95% Confidence Interval)
n = 25,689, deaths = 9,435, transplants = 767, censored = 15,487	
Model 1	1.15 (1.01 – 1.31)
Model 2	1.10 (0.96 – 1.25)

Model 1: adjusted for age, age-squared, sex, race, ethnicity, year of dialysis initiation, pre-dialysis and nephrology care

Model 2: model 1 + incident dialysis modality, cause of kidney failure, incident vascular access, primary insurance, urban/rural, and comorbidities

© 2023 Novick TK et al. *JAMA Network Open*.

eTable 8. Sensitivity Analysis: Including Individuals Aged Older Than 85 Years

Model	Hazard Ratio of All-cause Mortality (95% Confidence Interval)
Overall, n = 26,955; deaths = 10,143, transplants = 767, censored = 16,045	
Model 1	1.15 (1.02 – 1.30)
Model 2	1.10 (0.97 – 1.25)
Age ≥75; n = 7,288, deaths = 3,574, transplants = 29, censored = 3,685	
Model 1	1.59 (1.19 – 2.12)
Model 2	1.66 (1.24 – 2.22)

Model 1: adjusted for age, age-squared

Model 2: model 1 + sex, race, ethnicity, year of dialysis initiation, pre-dialysis incident dialysis modality, cause of kidney failure, incident vascular access, primary insurance, urban/rural, and comorbidities

© 2023 Novick TK et al. *JAMA Network Open*.

eTable 9. Sensitivity Analysis: Analysis With Age as a Categorical Variable

Model	Sub-distribution Hazard Ratio of All-cause Mortality for Unstable Housing vs Stable Housing (95% Confidence Interval)
n = 25,689, deaths = 9,435, transplants = 767, censored = 15,487	
Model 1	
<50 years	1.13 (0.51 – 2.47)
50-64 years	0.99 (0.83 – 1.18)
65-74 years	1.17 (0.94 – 1.47)
≥75 years	1.54 (1.12 – 2.10)
Model 2	
<50 years	1.06 (0.49 – 2.27)
50-64 years	0.94 (0.79 – 1.13)
65-74 years	1.15 (0.92 – 1.44)
≥75 years	1.54 (1.13 – 2.10)

Model 1: adjusted for sex, race, ethnicity, year of dialysis initiation, pre-dialysis nephrology care, and age group × unstable housing interaction term

Model 2: model 1 + incident dialysis modality, cause of kidney failure, incident vascular access, primary insurance, urban/rural, and comorbidities

© 2023 Novick TK et al. *JAMA Network Open*.

eTable 10. Sensitivity Analysis: Sample Limited to Participants Who Did Not Have Discrepancies Between US Renal Data System and Veterans Health Administration Data on Date of Death

Age Group	Sub-distribution Hazard Ratios of All-cause Mortality for Unstable Housing vs Stable Housing (95% Confidence Interval)	
n = 25,617, deaths = 9,363, transplants = 767, censored = 15,487		
	Model 1	Model 2
At median age (68)	1.24 (1.08 – 1.42)	1.19 (1.04 – 1.37)
Age = 40	0.74 (0.49 – 1.10)	0.67 (0.45 – 1.01)
Age = 45	0.81 (0.58 – 1.12)	0.75 (0.54 – 1.04)
Age = 50	0.89 (0.68 – 1.15)	0.83 (0.64 – 1.07)
Age = 55	0.97 (0.80 – 1.18)	0.92 (0.75 – 1.12)
Age = 60	1.07 (0.92 – 1.24)	1.01 (0.87 – 1.18)
Age = 65	1.17 (1.03 – 1.33)	1.12 (0.99 – 1.28)
Age = 70	1.29 (1.10 – 1.50)	1.24 (1.07 – 1.45)
Age = 75	1.41 (1.15 – 1.73)	1.38 (1.12 – 1.69)
Age = 80	1.55 (1.18 – 2.03)	1.53 (1.16 – 2.00)

Model 1: adjusted for age, age-squared, sex, race, ethnicity, year of dialysis initiation, pre-dialysis nephrology care, and age × unstable housing interaction term
Model 2: model 1 + incident dialysis modality, cause of kidney failure, incident vascular access, primary insurance, urban/rural, and comorbidities

© 2023 Novick TK et al. *JAMA Network Open*.

eTable 11. Sensitivity Analysis: Sample Limited to Participants Who Were Screened for Unstable Housing Within 1 Year Before Dialysis Initiation

Age Group	Sub-distribution Hazard Ratio of All-cause Mortality for Unstable Housing vs Stable Housing (95% Confidence Interval)	
n = 23,644, deaths = 8,373, transplants = 708, censored = 14,563		
	Model 1	Model 2
At median age (68)	1.27 (1.03 – 1.57)	1.24 (1.01 – 1.53)
Age = 40	0.92 (0.54 – 1.58)	0.86 (0.50 – 1.47)
Age = 45	0.98 (0.63 – 1.52)	0.92 (0.59 – 1.42)
Age = 50	1.03 (0.73 – 1.46)	0.98 (0.70 – 1.38)
Age = 55	1.10 (0.85 – 1.42)	1.05 (0.81 – 1.35)
Age = 60	1.16 (0.95 – 1.41)	1.12 (0.92 – 1.36)
Age = 65	1.23 (1.02 – 1.48)	1.19 (0.99 – 1.44)
Age = 70	1.30 (1.03 – 1.64)	1.27 (1.01 – 1.61)
Age = 75	1.38 (1.01 – 1.89)	1.36 (1.00 – 1.86)
Age = 80	1.46 (0.97 – 2.19)	1.45 (0.97 – 2.18)

Model 1: adjusted for age, age-squared, sex, race, ethnicity, year of dialysis initiation, pre-dialysis nephrology care, and age × unstable housing interaction term
Model 2: model 1 + incident dialysis modality, cause of kidney failure, incident vascular access, primary insurance, urban/rural, and comorbidities
Note: There were 771 with unstable housing in the original analysis (within 3-years of dialysis initiation), and 356 of those had a positive housing screen within the year before starting dialysis.

© 2023 Novick TK et al. *JAMA Network Open*.
