# Supplementary material for: Unstable Housing and Mortality Among US Veterans Receiving Dialysis
Source: JAMA Netw Open. 2023 Nov 21;6(11):e2344448. doi: 10.1001/jamanetworkopen.2023.44448 (PMC10663965; doi:10.1001/jamanetworkopen.2023.44448)
Supplement: Supplement 2. — Data Sharing Statement [file jamanetwopen-e2344448-s002.pdf]

Data Sharing Statement

Novick. Unstable Housing and Mortality Among US Veterans Receiving Dialysis. *JAMA Netw Open*. Published November 21, 2023. doi:10.1001/jamanetworkopen.2023.44448

Data
Data available: No
